# DISPARITIES IN ACCESS TO DENTAL CARE IN COMMUNITY-DWELLING OLDER ADULTS WITH COGNITIVE DISABILITY

**DOI:** 10.1093/geroni/igac059.1100

**Published:** 2022-12-20

**Authors:** Preeti Zanwar, Elizabeth Wood, Gilbert Gimm

**Affiliations:** Jefferson College of Population Health, Philadelphia, Pennsylvania, United States; Washington State University, Spokane, Washington, United States; George Mason University, Vienna, Virginia, United States

## Abstract

Older adults with cognitive disability are worse off with being up-to-date with preventive care and routine dental care is not considered a clinical preventive visit covered by insurance for this population. I examine disparities in access to dental and clinical preventive services (e.g. dental visit, blood pressure visit, flu shot) in the past year in 28,068 older adults with cognitive disability using the nationally representative Medical Expenditure Panel Survey from 2009-2016. I conduct multivariate logistic regressions and find older adults with cognitive disability vs. no disability have higher odds of receiving annual blood pressure check (AOR, 1.97, 95% CI 1.34-2.88) but lower odds for having an annual dentist visit (AOR, 0.61, 95% CI 0.53-0.71) with only one-tenth of those with cognitive disability report having dental insurance. These findings have implications for integrated community-and-clinical care partnerships for closing the gap for routine dental care services among older adults with cognitive disability.

